# Effect of preputial washing on bacterial load and preservability of semen in Murrah buffalo bulls

**DOI:** 10.14202/vetworld.2015.798-803

**Published:** 2015-06-28

**Authors:** G. S. Meena, V. S. Raina, A. K. Gupta, T. K. Mohanty, M. Bhakat, M. Abdullah, R. Bishist

**Affiliations:** 1 Krishi Vigyan Kendra, Bundi-323001, Rajasthan, India; 2 Artificial Breeding Research Centre, ICAR-National Dairy Research Institute, Karnal - 132 001, Haryana, India; 3 Dr. Yashwant Singh Parmar University of Horticulture & Forestry, Nauni, Solan-173230, Himachal Pradesh, India

**Keywords:** preputial washing, bacterial load, semen quality, preservability, Murrah buffalo bull

## Abstract

**Aim:**

To study the effect of preputial washing on bacterial load, preservability and semen quality in Murrah buffalo bulls

**Materials and Methods:**

A total of 36 collections of three Murrah buffalo bulls maintained at Artificial Breeding Research Centre, ICAR-National Dairy Research Institute, Karnal, were collected at weekly intervals from each bull without preputial washing and latter ejaculates from same bull with preputial washing by infusing normal saline (0.85%), KMnO_4_ (0.02%) and savlon (2.0%) to first, second and third bull, respectively. The microbial load and semen quality were evaluated during different hours of storage at refrigerated temperature (0, 24 and 48 h) and after thrawing of cryopreserved (at −196°C) semen.

**Results:**

The results of preservation of semen at refrigerated temperature showed that bacterial load was markedly lower in ejaculates of bulls subjected to preputial washing. Semen preserved at refrigerator temperature and cryopreserved, the effect of washing solution was significant for individual motility (IM), non-eosiniphilic count, hypo-osmotic swelling reactivity (HOST), total plate count (TPC) and acrosome integrity. KMnO_4_ was found to be the best in lowering bacterial load, sperm abnormalities and in improving semen quality such as motility, non-eosinophilic count, HOST and acrosome integrity even up to 48 h of preservation and cryopreserved semen. Effect of duration of preservation and stage of cryopreservation was also significant for IM, non-eosiniphilic count, HOST, sperm abnormalities and acrosome integrity.

**Conclusion:**

Overall the results suggested that preputial washing with KMnO_4_ solution improved the semen quality and reduced microbial load of Murrah buffalo bull’s semen preserved at refrigerated temperature and cryopreservation.

## Introduction

Production and dissemination of disease free quality semen are the major thrust of semen stations in India and throughout the world. Most of semen stations in India strictly follow minimum standard protocol for frozen semen production through diseases testing at regular interval to avoid the spread of the pathogen and widespread of ubiquitous bacteria by contaminated semen. The success of artificial insemination (AI) depends on the quality semen production and supply. One of the important factors, which influence semen quality [1] and further fertility [2] is a bacterial load in semen, therefore, considered in the quality control of semen [3]. In the breeding season, the sexual activity may lead to contamination of the penis and prepuce [4], which might increase microbial load in semen in natural mating condition. Higher microbial load in semen is a reflection of unhygienic management in various steps of bull management, semen collection, and processing [5]. To use semen for AI for satisfactory conception [6], the bacterial load should not be more than 5000 Cfu/dose.

Microbial contamination has effect on motility, morphology and various semen quality parameters [7] which may be due to direct effect or competition for nutrients [8] detected aerobic bacteria in almost all the collected semen samples but the various opportunistic pathogenic organisms in semen may cause reproductive disorders as well as may result in lower conception rate and increase in embryonic mortality, abortion and other complications in female. During bacterial isolation studies many types of bacteria have been found in frozen semen [9]. Bacterial contamination in frozen semen first leads to the production of macrophages and polymorphonuclear granulocytes and these cells generate reactive oxygen species that in turn impair sperm function and reduces its fertilization capability [10].

Many semen banks in India are getting microbial load estimated in frozen semen doses by either identified laboratory or from outside agencies or by themselves regularly [11]. Highly pathogenic bacteria like *Tritrichomonas foetus* inhabit the penis and prepuce of bull [12] as well as aerobic bacteria have been detected in almost all the semen samples collected from rams [13] and buffalo bulls [14], therefore though it is very difficult to achieve microbe free semen production but it can be minimized by routine preputial washing and hygienic bull management. Washing of preputial cavity prior to the collection of semen has therefore been advocated by many investigators to prevent microbial contamination. Very scanty information is available regarding impact of preputial washing on semen quality at refrigeration temperature and cryopreservation in Murrah buffalo besides the well-established fact of decrease in microbial load after preputial washing (APW) in crossbred and exotic bulls [15-17]. With an overall objective to augment quality germ plasm production in Murrah buffaloes, the present investigation was therefore carried out to study the effect of prepuce washing on semen quality including microbial quality aspects.

## Materials and Methods

### Ethical approval

We have carried out the experiment in accordance with the Guidelines laid down by the Institutional ethics committee of ICAR-NDRI with prior approval.

### Animals

The present investigation was conducted on three sexually healthy Murrah buffalo bulls maintained at Artificial Breeding Research Centre, ICAR-National Dairy Research Institute (ICAR-NDRI), Karnal.

### Study area

The farm is located on 29° 42′N latitude and 72° 02′E longitude at an altitude of 250 m above the mean sea level in the bed of Indo-Gangetic alluvial plain. A subtropical climate prevails in the area. There are four major seasons i.e. Winter, Summer, Rainy and Autumn in the year.

### Management of bulls

Bulls were kept in the individual bull pen (30′×10′) separated by solid partitions that restricted both direct physical and visual contact of bulls in adjacent pens as well as free movements within the shed. Bulls were exercised once a week, the day before semen collection in the rotary exerciser so as to maintain the sexual vigor of bulls and ensure quality semen production. Vaccination, deworming. Screening of sexually transmitted disease and other herd-health program was followed as per the farm schedule, to ensure good health.

### Preputial washing

The preputial washing was done by infusing sterile, warm (37°C) solutions of normal saline (0.85%), KMnO_4_ (0.02%) and savlon (2.0%) to first, second and third bull, respectively. The solution (100 ml/bull) was infused with the help of a sterile disposable plastic syringe (60 ml) and white sheath of AI gun just 10 min prior to semen collection.

### Semen collection

Semen was collected twice a week from each bull using sterilized artificial vagina. Two ejaculates were taken from a bull at an interval of at least 30 min in the morning hours. A total of 36 collections comprising of first ejaculate from each bull without preputial washing and latter ejaculates from each bull with preputial washing were taken.

### Semen quality assessment

Immediately after collection, the ejaculates were brought to the laboratory and kept in water bath at 30°C for assessing volume, color, mass-activity, individual motility (IM), sperm concentration, non-eosinophilic, hypo-osmotic swelling test and acrosome integrity for subsequent experimentation. Semen was diluted in tris egg yolk extender to conduct studies on preservability of semen. Split semen samples were further processed for preservation at refrigerator temperature (4°C) and for cryopreservation at −196°C in liquid nitrogen. The microbial load and sperm quality was evaluated at different hours of storage at refrigeration temperature (0, 24 and 48 h) as well as before and after freezing of semen. The bacterial count/ml of the sample were estimated by multiplying the dilution factor with the mean number of colonies in media plates (Bacterial load/ml=Bacterial colonies counted on plate × dilution factor).

### Statistical analysis

Data were analyzed using two-way ANOVA with interaction. The percent data on semen characteristics were subjected to arcsine transformation and a microbial count was subjected to log transformation for analysis [18].

## Results

Artificial vagina (AV), glassware, semen dilutor and materials coming in contact with semen during processing are contributing to the bacterial load of the semen. Therefore, AV, glassware and extender used were subjected to assessment of bacterial load. The standard procedure of sterilization was followed to get contamination free AV. The average values of bacterial load (cfu) from washing of AV just before and after collection were 415 and 76000, respectively. Glasswares which were subjected to contact with semen during processing and handling, were sterilized by hot air oven. The average bacterial load (cfu/ml) of sterilized and un-sterilized glassware was 112 and 42000, respectively. The average bacterial load (cfu/ml) of Tris egg yolk extender without any antibiotic treatment was 380.42 (cfu/ml). The average bacterial load (cfu/ml) in preputial washings by saline, savlon and KMnO_4_ were 3187.17, 2536.33 and 2292.83, respectively. Semen samples were processed for the preservation of semen at 4°C and −196°C and semen quality was assessed.

### Preservation of semen at refrigeration temperature (4°C)

Least squares means of percent individual sperm motility, non-eosinophilic count (live), hypo-osmotic swelling (HOS) reacted spermatozoa and total plate counts (TPCs) in semen samples before and APW at 0, 24 and 48 h of preservation at refrigerated temperature are presented in Table-1. The average (%) individual sperm motility, non-eosinophlic (live), sperm abnormalities, intact acrosome, hypo-osmotic swelling reactivity (HOST) reacted spermatozoa and microbial load estimates of semen collected before prepuce washing (BPW) of bulls and preserved at 4°C for 0, 24 and 48 h are presented in Table-1.

**Table-1:** Semen characteristics and bacterial load (×10) in semen stored at refrigerated temperature (up to 48 h) obtained before and after preputial washing of Murrah bulls.

Groups	0 h						24 h						48 h					
		
	IM	N-eosin	SPA	IA	HOST	TPC	IM	N-eosin	SPA	IA	HOST	TPC	IM	N-eosin	SPA	IA	HOST	TPC
BPW (control)																		
M	69.72^a^	78.78^a^	6.33	83.17^a^	61.72	114.28^a^	61.11	64.11^a^	13.22^a^	73.83^a^	54.50	83.56^a^	49.44	57.89^a^	15.00	55.17	43.89	78.72
SE	2.08	1.03	0.40	2.18	1.74	22.73	2.20	1.42	1.07	2.27	1.99	13.92	2.02	1.77	1.28	1.76	1.72	19.44
APW (treatments)																		
Saline																		
M	71.67^ab^	80.33^a^	6.50	90.67^ab^	63.67	43.00^ab^	64.17	68.00^a^	8.50^b^	76.33^ab^	57.83	36.17^ab^	52.50	62.33^a^	12.17	57.67	45.33	32.17
SE	2.47	1.48	0.43	1.26	2.33	11.37	2.01	1.55	0.43	2.51	1.68	4.19	1.71	2.94	0.48	2.40	1.89	5.12
Savlon																		
M	77.50^ab^	88.00^b^	5.50	86.83^ab^	65.50	35.50^ab^	65.83	76.33^b^	8.67^b^	76.50^ab^	58.83	32.83^ab^	53.33	64.33^b^	12.33	60.67	46.83	28.50
SE	3.35	3.68	0.67	4.06	3.12	6.01	3.75	3.77	1.43	4.06	3.71	8.32	4.01	2.39	0.95	8.58	3.94	4.17
KMnO_4_																		
M	80.00^b^	89.50^b^	5.00	93.50^b^	66.83	26.50^b^	69.17	81.67^b^	7.17^b^	82.14^b^	62.17	24.83^b^	57.50	67.33^b^	11.17	66.67	51.00	21.67
SE	3.42	1.50	0.00	0.62	1.42	4.50	2.71	1.73	0.79	1.53	2.48	8.34	1.71	1.91	0.60	3.60	1.67	5.64

BPW=Before preputial washing, APW=After preputial washing, M=Mean, SE=Standard error, IM=Individual sperm motility (%), N-eosin=Non-eosiniphilic count (%), SPA=Total sperm abnormalities (%), HOST=Hypo-osmotic swelling reactivity (%), IA=Intact acrosome (%), TPC=Total plate count (cfu/ml). Values with different superscripts within a column differ significantly (p<0.05)

At 0 h IM at 4°C was significantly higher in case of preputial washing with KMnO_4_ as compared to BPW group. After 24 and 48 h of preservation, there was no significant difference in motility between BPW and APW groups. At 0, 24 and 48 h of preservation, the non-eosinophilic sperm values (%) were significantly higher in APW groups except for saline treated bull. Preputial washing with KMnO_4_ resulted in a highest percentage of non-eosinophilic spermatozoa, followed by savlon and saline groups, respectively. After 0 and 48 h of preservation, there was no significant difference in the values of total sperm abnormalities between APW and BPW group. After 24 h of preservation, the values of total sperm abnormalities were significantly higher in BPW group than APW group. In APW group the abnormalities were lowest in KMnO_4_ followed by savlon and saline. However, there was no significant difference in all three solutions. After 0 and 24 h of preservation significant differences were observed between KMnO_4_ and other treatment groups, as well as between APW and BPW groups. The percent values of HOST in prepuce washing groups were higher than these without prepuce washing group. No significant difference in HOST percent between BPW and APW groups was observed. Highest HOST positive spermatozoa were observed in KMnO_4_ treated group followed by savlon and saline. Microbial load in semen was the lowest in case of prepuce washing with KMnO_4_ solution till 24 h of preservation. After 48 h of preservation, there was no significant difference in microbial load between BPW and APW groups. The bacterial load in refrigerated semen in the present study was significantly lower in APW group than BPW group.

### Cryopreserved semen

Least squares means of individual sperm motility, percent non-eosinophilic counts, HOST reacted spermatozoa and TPCs in semen samples before and APW and subjected to cryopreservation are presented in Table-2. The average IM, non-eosinophlic (live); sperm abnormalities; intact acrosome; HOST reacted spermatozoa and microbial load values in pre-freeze and post-thaw semen are presented in Table-2.

**Table-2:** Semen characteristics and bacterial load (×10) in cryopreserved semen collected BPW and APW.

Groups	Pre freeze semen						Post thaw semen					
	
	IM	N-eosin	SPA	IA	HOST	TPC	IM	N-eosin	SPA	IA	HOST	TPC
BPW (control)												
M	69.72^a^	78.78^a^	6.33	83.17^a^	61.72	114.28^a^	48.33	61.50	15.17	62.56^a^	37.89^a^	65.61
SE	2.08	1.03	0.40	2.18	1.74	22.73	1.57	1.58	1.09	1.26	1.58	16.04
APW (treatments)												
Saline												
M	71.67^ab^	80.33^a^	6.50	90.67^ab^	63.67	43.00^ab^	52.50	64.17	13.83	64.33^ab^	40.33^ab^	23.17
SE	2.47	1.48	0.43	1.26	2.33	11.37	2.14	2.01	2.20	2.03	3.88	4.81
Savlon												
M	77.50^ab^	88.00^b^	5.50	86.83^ab^	65.50	35.50^ab^	54.17	66.33	12.67	69.00^ab^	44.17^ab^	21.17
SE	3.35	3.68	0.67	4.06	3.12	6.01	5.07	5.09	1.45	4.09	4.47	4.53
KMnO_4_												
M	80.00^b^	89.50^b^	5.00	93.50^b^	66.83	26.50^b^	55.00	67.67	11.83	73.67^b^	50.33^b^	16.67
SE	3.42	1.50	0.00	0.62	1.42	4.50	2.89	2.50	2.15	2.40	4.77	5.19

BPW=Before preputial washing, APW=After preputial washing, M=Mean, SE=Standard error, IM=Individual sperm motility (%), N-eosin=Non-eosiniphilic count (%), SPA=Total sperm abnormalities (%), HOST=Hypo-osmotic swelling reactivity (%), IA=Intact acrosome (%), TPC=Total plate count (cfu/ml). Values with different superscripts within a column differ significantly

In pre-freeze and post thawed semen the motility values were significantly higher for KMnO_4_ treated bull semen as compared to saline, savlon and BPW groups. In post thawed semen, the motility was not significantly higher in APW group than BPW group. In pre-freeze semen non eosinophilic sperm values were significantly higher in KMnO_4_ and savlon treated groups. Whereas, in post thawed semen there was no significant difference among different treatments and solutions used. Preputial washing with KMnO_4_ resulted in lowest sperm abnormalities. In pre-freeze semen no significant difference in total sperm abnormalities was observed between BPW and APW group. In pre-freeze and post-thaw semen intact acrosomes were significantly higher in KMnO_4_ as compared to BPW, savlon and saline treated bull semen samples. This may be due reduction of toxic metabolites of bacteria and thus plasma membrane integrity remains intact in KMnO_4_ treated group. Post thawed semen HOST values were significantly higher in KMnO_4_ treated semen samples. The analysis of variance showed significant effect (p<0.01) of stage, solution and stage-solution interaction on HOST. The HOS response and acrosome status of sperms in APW group were significantly higher than BPW group. Prepuce washing with KMnO_4_ treatment had the highest efficacy in terms of sustaining membrane integrity. In pre-freeze stage, bacterial load was significantly less in semen samples APWs with KMnO_4_ solution than the samples without preputial washing. In post thawed semen there was no significant difference in microbial load among different treatments.

## Discussion

The findings of bacterial load in refrigerated semen after preputial wash in the present study were similar to the results of various researchers in crossbred bull [15] and in camel [19]. Microbial contamination in semen may interfere with the sperm function and fertilization potential due to the formation of reactive oxygen species by macrophages and polymorphonuclear granulocytes and decrease in motility due to adherence of microbes with spermatozoa. Microbes may have a direct effect on acrosome through toxin production [10]. Bacteria appear to exert their spermicidal effect directly upon the sperm cell. Bacteria compete with cells (spermatozoa) for nutrients and oxygen necessary for growth and normal functioning [20]. The preputial cavity is the main source of contamination in semen. Most of the bacteria can be declared as a commensal, but there are also potentially pathogenic germs that can contaminate the collected semen [21]. In semen *Staphylococcus* spp., *Escherichia coli*, *Streptococcus* spp. are the most common organisms [22], which may have an influence on the motility and viability of bovine semen. Preputial cavity is the main source of contamination in semen. Therefore, preputial washing with antiseptic solution helps in reduction of microbial load, which resulted in better quality semen by inactivating or destroying microbes in semen.

The results of non-eosinophilic sperm values were in accordance with those of findings earlier reported by Bindra et al. [16]. This result may be due to minimum bacterial contamination and improvement in hygienic quality of semen before freezing. The HOST and acrosome status of sperms in semen are in conformity with those of Monga and Roberts [23]. Similar to our results Ortega-Ferrusola et al. [24] concluded that the microbial flora of the ejaculate may be responsible for damage of spermatozoa during cryopreservation. Higher sperm abnormalities in BPW group might be due to spermicidal and deleterious effect of bacteria. Reports depict that sometime morphological abnormalities leads to changes in the acrosomal integrity of spermatozoa [25]. The sperm abnormalities in semen were the lowest in KMnO_4_ treated bull semen. It may be due to the better efficacy of the solution against various organisms. The microbial load among post thawed semen in different treatments showed findings akin with those of Bhakat and Raina [17]. Initial quality of semen can be improved hygienically by adopting preputial washing before semen collection.

The better efficiency of KMnO_4_ for preputial washing as compared to savlon and saline solution may be due to effective control of various microorganisms (bacteria, fungi, virus and algae), on the other side savlon acts as bacteriostat and effectivity of saline is further less. KMnO_4_ also helps in reduction of toxins produced by microorganisms, which further helps in reduction of oxidative stress on spermatozoa, thus showing in the improvement of semen quality. The broad spectrum role of KMnO_4_ may help to control and inactivate microbes and shows more effective as compared to savlon and normal saline. It may be due to minimum bacterial contamination and improvement in hygienic quality of semen before freezing.

The process of freezing has great impact on motility, sperm abnormalities, normal acrosomes and intact plasma membranes and in similar fashion the quality is getting affected during preservation of semen at refrigeration temperature, but severity may be less, as there is increase in large numbers of dead and abnormal sperms after cryopreservation as compared to preservation at refrigerated temperature. Dead spermatozoa normally release reactive oxygen species (ROS) and certain other toxic substances. ROS acts through lipid peroxidation of carbon chain of unsaturated fatty acid to form highly cytotoxic lipid hydroperoxides, which decompose to form end product malondialdehyde, which is highly toxic and is responsible for DNA and protein damage finally leading to cell death as well as damage the fertilization potential of other healthy sperms. During cryopreservation and thawing process there was considerable damage to the motility apparatus, plasma membrane and acrosomal cap which may be due to leakage of enzymes responsible for sperm motility, weaker plasma membrane of spermatozoa, dehydration, ice-crystal formation during freezing as well swelling and corrugation of the anterior part of the acrosome. There was considerable irreversible loss after cryopreservation, which cannot be avoided.

## Conclusion

Overview of the results suggested that prepuce washing with KMnO_4_ solution would facilitate quality semen production in terms of reduced microbial load, sperm abnormalities and higher sperm motility, livability, membrane integrity, acrosome intactness in the semen stored at refrigerator temperature (4°C) and in liquid nitrogen (−196°C) due to broad spectrum effect. Use of a higher amount of KMnO_4_ for preputial washing is not advisable as it may act as an irritant, which may affect the quality semen production. Preputial washing with normal saline and savlon is also good, but best results can be obtained using KMnO_4_ for prepuce washing.

## Authors’ Contributions

GS did bench work under the supervision of VSR, AKG and TKM. MB, RB and MA helped in the technical input, bench work, statistical analysis and in making the final manuscript of the research. All authors read and approved the final manuscript.
